# Evaluation of the Waste Tire Resources Recovery Program and Environmental Health Policy in Taiwan

**DOI:** 10.3390/ijerph6031075

**Published:** 2009-03-12

**Authors:** Chia-Ching Chen, Tetsuji Yamada, I-Ming Chiu, Yi-Kuen Liu

**Affiliations:** 1 New York Medical College School of Public Health, 95 Grasslands Road, Valhalla, New York 10595, USA; 2 Rutgers University, the State University of New Jersey, 311 North Fifth Street, Camden, New Jersey 08102, USA; E-Mails: tyamada@crab.rutgers.edu (T.Y.); ichiu@camden.rutgers.edu (I-M.C.); 3 Environmental Protection Administration (EPA) of Taiwan, No.83, Sec. 1, Jhonghua Rd., Jhongjheng District, Taipei City 100, Taiwan; E-Mail: ykliu@sun.epa.gov.tw; 4 Department of Public Health College of Medicine, Fu-Jen Catholic University, No.510, Jhongjheng Rd., Sinjhuang City, Taipei County 24205

**Keywords:** Environmental health policy, waste tires, health outcome

## Abstract

This paper examines the effectiveness of Taiwanese environmental health policies, whose aim is to improve environmental quality by reducing tire waste via the Tire Resource Recovery Program. The results confirm that implemented environmental health policies improve the overall health of the population (i.e. a decrease in death caused by bronchitis and other respiratory diseases). Current policy expenditures are far below the optimal level, as it is estimated that a ten percent increase in the subsidy would decrease the number of deaths caused by bronchitis and other respiratory diseases by 0.58% per county/city per year on average.

## 1. Introduction

Many epidemiological studies have suggested that there is a significant and direct correlation between air pollution and certain respiratory diseases such as bronchitis, asthma, emphysema, and lung cancer. Respiratory diseases directly attributable to air pollution are the second largest cause of premature death in Taiwan. Local Taiwanese communities are aware of this problem and often blame the activities of tire-recycling companies for creating a polluted and unhealthy environment. Government subsidies provided through environmental health policies aid in the abatement of hazardous environmental conditions detrimental to the populations’ health via the resource recovery program sponsored by the Taiwanese Environmental Protection Administration (EPA). These efforts result in overall better health outcomes for the population [1]. The resource recovery program is encouraged not only from the perspective of recycling tires for use as a resource, but also from an environmental protection perspective of improving the quality of air, soil and water. The lack of effective regulation of tire waste leads to non-standardized methods of storage, decomposition and disposal practices for tire waste, which in turn influences environmental pollution. Recycling companies handling the various phases of scrap tire disposal (i.e. collection, storage, transport, decomposition, and disposal) have been booming as they are highly profitable. Waste tires accumulated for the purpose of resource recovery at tire recycling factories/sites while left exposed to the elements will continually deteriorate environmental quality. Horner analyzed the pollution resulting from the decomposition of waste tires and found heavy metals (cadmium, lead. and zinc) leaking into soil [2]. The environmental pollution caused by accumulated waste tires at these locations is considered an “environmental hazard stressor” [3]. Scrap and waste tire piles are non-treated hazardous materials. However, if a fire occurs, tires break down into hazardous compounds including toxic gases, heavy metals, and a flammable oil compound. These tire fires results in thick smoke throughout the surrounding area that contains pollutants harmful to human health. An increase in this stressor, which is defined as an internal and external environmental change, may upset an individual’s physical and psychological well-being.

The unmanaged activity of tire recycling creates social costs. In order to arrive at a healthier set of outcomes, the environmental health policies of the Taiwanese government and tire-recycling activities must be examined. Environmental health hazards that increase health risks cause various respiratory diseases which may result in death. Considering the social costs involved, it is necessary to determine the optimal amount of subsidy to effectively reduce the environmental health risks posed by the tire recycling industry. Schwartz underscored the effects of suspended particulates (TSP) on mortality excluding accidental and violent causes in the U.S.A. [4,5]. Lee *et al.* emphasized the effects of TSP, ozone (O_3_), and sulfur dioxide (SO_2_) on mortality in Korea with a definition similar to that of Schwartz’s [6]. O’Neill, Loomis and Borja-Aburto underlined the effects of ozone and PM_10_ on mortality that excluded accidental causes in Mexico [7]. It is essential to distinguish the effect of the environmental health policies of the government from that of accumulated waste tires at tire recycling factory sites. They have different implications for environmental health policy and for policy guidance regarding tire-recycling activities. The net effect on population health outcomes stemming from the environment health policies as well as the accumulated waste tires at tire-recycling factories is ambiguous. While the idea of environmental health hazards posed by scrap and waste tires on health status is an increasingly important issue, few studies have examined how the governments’ environmental health policies and tire-recycling activities affect the overall health of the population (i.e., the numbers of deaths caused by respiratory disease).

This study hypothesizes that the government’s environmental health policy will improve the health outcomes of the population. However, accumulated waste tires waiting to be processed, lack of proper treatment and storage facilities along with the decomposition of waste tires may deteriorate environmental conditions and adversely affect the populations’ health. Additionally, illegal incineration often generates highly harmful pollutants. Although the waste tire resource program is small relative to the overall Taiwanese environmental protection program, ineffective guidance and management of the program creates an “environmental health hazard stressor.” This stressor deteriorates health and in the long term will affect respiratory diseases and death. Stressors in general are risk factors that produce health disparities, health morbidity, and incidence of illnesses (i.e. respiratory illnesses, premature mortality, *etc.*).

## 2. Literature Review

In spite of the apparent health risks posed by accumulated disposal and scrap tires, few studies have been conducted on the two issues regarding environmental health policy and the Taiwanese tire-recycling program. From an environmental management standpoint, the tire-recycling program is poorly enforced and administrative regulations themselves have become a source of environmental pollution. The Department and Bureau of Environmental Protection of Taiwanese Government subsidizes the tire recycling industry, approving procedures, locations and capacities of tire-recycling operations, etc. Incineration processes and stockpiles at factories/sites which pose fire hazards are a potential source of air pollution due to the inadequate administrative regulations and procedures and the shortage of recycling facilities. Horner warned of the potential environmental pollution produced by the disposal of used car tires and scrap tires in West London [2]. Chang *et al.* examined the association between levels of air pollutants and hospital admissions for cardiovascular diseases in Taipei from 1997–2001 [8]. They found a positive statistically significant effect of respirable particle pollutant (PM_10_), ozone (O_3_), nitrogen dioxide (NO_2_), and carbon monoxide (CO) on hospital admissions. Shaw and Hung evaluated the new air pollution control policy known as the cap-and-trade program, implemented in 1999 by the Taiwanese EPA [9]. They rigorously assessed air pollution control policies: command-and control, tax-allowance subsidy, emission fees, and cap-and-trade, in Taiwan. In addition to the emission fees collected from the importers and producers of ozone-depleting substances and from motorists, the EPA imposes a cap on aggregate emissions for credit trading because of the rapid growth of economic activities and the tremendous use of the various types of vehicles in Taiwan (See the detail policy issues in http://www.epa.gov.tw/b/b0100.asp, and http://www.epa.gov.tw/epalaw, July 20, 2007).

To examine the effects of short-term exposure to airborne particles, Braun-Fahrlander *et al.* conducted a study to investigate the association between air pollution and respiratory symptoms in two Swiss cities [10]. They concluded that the incidence and duration of respiratory symptoms are likely to be associated with airborne particulate concentrations as well as duration of exposure. The study results were consistent with those of Pope [11]. As for the effects of PM_10_ and O_3_, Gilliland *et al.* used a two-stage time-series model and found that a short-term change in O_3_ is associated with substantial increases in school absences [12]. Ho *et al.* focused on junior high school students in Taiwan and concluded that air pollution (i.e. PM_10_ and O_3_) was correlated to asthma attack rates [13].

Total suspended particulate matter (TSP) has been found to be associated with all chronic respiratory conditions according to the U.S. Health Interview Survey [14]. Archer showed that long-term exposure to TSP resulted in an increased risk of developing cancer [15]. Later research by Schwartz used a distributed lag model to evaluate the association of the deaths of people over the age of 65 on a daily basis that were exposed to PM_10_ in 10 U.S. cities [16]. The study concluded that a 2-day moving average is substantially better than a 1-day average in estimating the effects of PM_10_. Another study using the lag structure with a 2-day moving average by Braga *et al.* also found interesting results: respiratory deaths are affected more by the PM_10_level on previous days, whereas cardiovascular deaths are more affected by same-day pollution [17]. Kim *et al.* examined the effects of ozone on mortality rates while taking the lag influence into consideration in Seoul, Korea [18]. He found that ozone risk in the summer from 1995 through 1999 was more serious than during the other seasons. Kim *et al.* and O’Neill *et al.* excluded accidental, poisoning and violent causes of death from their mortality statistics in the International Classification of Diseases (ICD) 10^th^ revision [7,18]. O’Neill *et al.* focused on the relationship between O_3_ and the mortality rate from 1996 through 1998, taking 2 lags’ influence in Mexico City, and underscored that O_3_ was positively associated with the mortality rate [7]. O’Neill also concluded that the elderly were at a higher risk for ozone-associated mortality.

As for respiratory morbidity levels associated with respirable particle pollution (PM_10_) and ozone (O_3_), Choudhury *et al*. reported that the morbidity of asthma, bronchitis, and other upper respiratory infections is caused by PM_10_ [19]. Peters *et al.* found in a 10-year prospective cohort study that the air pollutants, PM_10_ and O_3_, led to an increased prevalence in schoolchildren’s respiratory morbidity [20]. Chay and Greenstone; and Currie and Neidell found a positive relationship between infant mortality and PM_10_ [21,22]. Most recently, Ho *et al.* underlined the positive effect of air pollution (i.e. PM_10_, O_3_, nitrogen dioxide, nitrogen oxides, *etc.*) on asthma attack rates among junior high school students [13]. They examined monthly asthma attack rates verses single air pollutant concentration and found that carbon monoxide and pollution standard index were not statistically significant, and that sulfur dioxide had a negative effect on asthma attack rates.

## 3. Background

In Taiwan, various waste materials generated by economic activities are recycled and reused. However, due to the high population density and scarce land resources, industrial waste as well as normal refuse are generated in large quantities. The first priority, therefore, lies in the proper processing of these waste and trash materials to reduce pollution. Twenty-one items including discarded motor vehicles, waste containers, and tires have been listed as harmful to the environment. They are harmful in that they contain substances that will not naturally degrade in the short-term and are difficult to recycle. Without proper handling, these materials will severely degrade the environment and impart a large social cost burden to future generations.

The tire-recycling program is economically crucial for effective resource utilization due to Taiwan’s limited natural resources. An incineration process has been selected as the primary mode of trash processing. In 1997, the Environmental Protection Agency (EPA) introduced the “Four-in-One Recycling System”. This system joins the people, recycling management companies, local governments and the Recycling Management Fund (RMF) to promote all aspects of waste recycling. In order to minimize air pollution, safeguard public health and improve the overall quality of life the EPA modified the Air Pollution Control Act in 1999. Since this time tires have been considered articles that should not be sent to incinerators or landfills, but instead should be extracted, collected and sent to recycling centers for treatment. Additionally the EPA has implemented regulations requiring proper management of waste tires in an effort to limit the practice of illegal dumping. Tire piles and dumps can be found in a wide variety of settings such as big cities, small towns, and the countryside, and cleaning up these illegal dump sites is both time consuming and expensive. The average recycling rate is approximately 60% of the authorized scrap vehicle collection companies.

The EPA’s Recycling Management Fund Committee finances the subsidy via a prepaid tire-recycling taxation fee charged to tire producers and importers. The tax charged depends on the size of tire: less than and equal to 10 in. (T$10, Taiwan Dollars denoted by T$); 12–14 in. (T$50); 15–19 in. (T$60); 20–23 in. (T$150); and more than or equal to 24 in. There is no 11in. tire size for the prepaid tire-recycling taxation fee by the EPA of the Taiwanese Government. Waste tire collection companies receive T$0.6/kg from tire-recycling companies, the funding for which comes primarily from the EPA. The tire-recycling company receives T$3.2/kg, where waste tires are collected within their authorized districts, or T$2.4/kg, for waste tires collected outside these districts. This money is provided by the EPA foundation once the machine-comminute powder process or pyrolysis (heat dissolving) has been completed.

For resource recovery and recycling products, waste tire management and methods of reuse are as follows: (1) the machine-comminute powder: After comminute processing of tires, the material can be added to raw materials in the production of new goods - construction materials and other rubber products (including cement brick, ground brick or related replacement products, rubber powder, recycling gum, and other related rubber products). Most of the tire-recycling companies in Taiwan operate with using the machine-comminute powder method; (2) pyrolysis (heat dissolving): When there is heat and no oxygen, tires dissolve into oil products, carbon and flammable gas, as well as resources for recycling steel. In Taiwan, many factories and companies do not utilize the pyrolysis process because of the high capital investment required and the high operating costs inhibit the pyrolysis process from being commercially available; (3) auxiliary fuel: This recycling method utilizes raw energy in cement factory kilns, gas-and-electricity symbiosis facilities in power stations as well as paper factories in their steam equipment. For those usages that do not require extra work, tires can be used in various places, including artificial rocks, civil engineering, tunnel engineering, wharf engineering, agricultural use, and entertainment facilities (Policy Plan for the Tire-recycling Management Cost, the EPA of Taiwanese Government in 1999 and in 2005). There are many privately owned small-scale factories and designated large-scale factories.

## 4. Method

### 4.1. Analytical Framework: Application of the Transactional Model

The government encourages tire-collection and recycling companies to recycle waste tires. These companies accumulate waste tires for the purpose of resource recovery at factories and sites. The uncollected scrap and waste tires at these facilities normally generates harmful air pollution that adversely affects people’s health. The average recycling rate is 60% which is low. We note that the major sources of air pollution in Taiwan are heavy industries and petro-chemical industries. Evans and Cohen describe “stressors” as demands made by the internal or external environment which upset an individuals’ homeostasis. This in turn, affects their physical and psychological well-being which requires action to restore balance or equilibrium [23]. In the 1960s and 1970s, stress was considered to be a transactional phenomenon. The central concept of stress is that a given event or situation may be perceived in different ways by different individuals, and these subjective perceptions are the main determinants of effects on subsequent behavior and health status [3,24]. Therefore, environmental health hazards are a stressor for respiratory diseases; respiratory disease is one of the risk factors for premature death in Taiwan. The Transactional Model of Stress and Coping provides a useful framework for evaluating the processes of stressful events [3,25]. Glanz states that stressful experiences are constructed as person-environment transactions wherein the impact of an external stressor is mediated by the person’s appraisals of the particular stressor and the psychological, social, and cultural resources [24].

**Figure 1.**
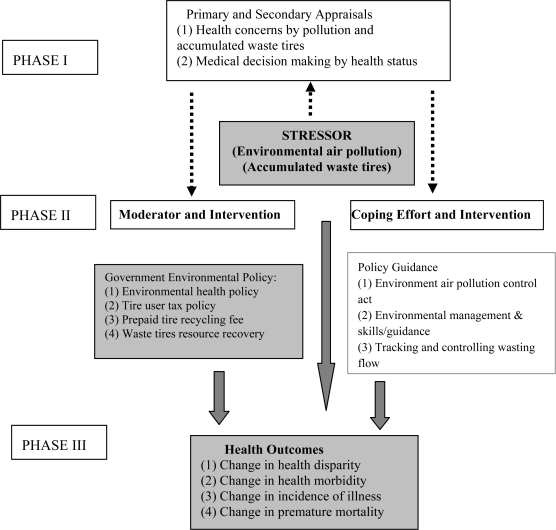
Application of the Transactional Model to Environmental Health Hazard, Government Health Policy, and Health Outcomes. Source: Glanz, K.; Rimer, B.K.; Lewis. F.M. Theory, research, and practice in health behavior and health education. In *Health behavior and health education*; Glanz, K., Rimer, B.K., Lewis, F.M., Eds.; Jossey-Bass: San Francisco, USA, 2002.

Appling the Transactional Model to Environmental Health Hazard, Government Health Policy, and Health Outcomes (Figure 1) shows that when a person faces a stressor such as an environmental health hazard, i.e. air pollution and accumulated waste tires, that person realizes his or her susceptibility and perception to them and the severity of the stressors, leading them to evaluate their potential threat or harm (primary appraisal) [3]. Thus, environmental air pollution and accumulated waste tires directly affect health. In the second appraisal phase, the degree of personal controllability of a stressor is attributed to an individual’s perceived ability to alter the situation, and manage the negative emotional reactions toward the stressor. As a moderator, the government’s environmental health policy directly influences health outcomes, independently of the stressor. The purposes of the government’s environment policy and policy guidance are to not only to reduce air pollution to increase the quality of health, but also to encourage resource recovery from waste tires. Management of health problems (i.e. psychological well-being, functional status, and health behavior), alleviates environmental hazards through government intervention [23].

In spite of the tire recycling program, efforts to moderate and mitigate stressors that cause health deterioration and disparities are questioned, as seen in phase II. Thus, applying the transactional model to environmental hazards and air pollution allows us to evaluate the effects of the environmental health policy as well as examine the government’s intervention in a systematic manner. The effects of the stressor and the policy process depend on the context (i.e. the mitigation of stressors harmful to human health, timing (short- versus long-term effects of environmental hazards and air pollution of adopting a government health policy), and characteristics). Coping efforts, which are beyond the scope of this research, are aimed at the environment air pollution control act, problem management/skills and guidance for tire-recycling companies, and tracking and controlling waste flow under the government policy guidance.

**Figure 2.**
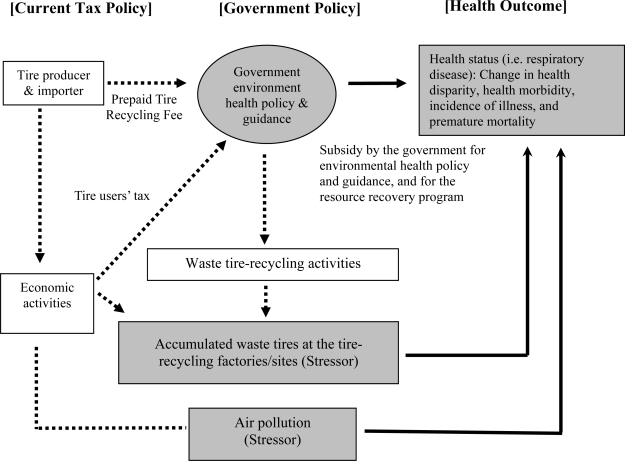
Flowchart of Environmental Health Policy and Resource Recovery Program: A Case in Taiwan. *Note*: The bold line is directly associated with health of population that this study focus on. A stressor is a function of accumulated waste tires at the tire-recycling factories/sites and air pollution.

The purpose of the study is to evaluate the effects of the government’s environmental health policy, accumulated waste tires, and air pollution on health. The net effect of the environmental health policy and the accumulated waste tires by tire-recycling factories and sites on the health outcome is ambiguous. Tire-recycling activities are indirectly related to the health of the population, and accumulated waste tires at factories and sites are directly related to environmental health hazards as seen in Figure 2. This life threatening stressor causes numerous premature deaths that are currently overlooked by policymakers.

The stressor creates a social cost incurred by environmental health hazards and air pollution [26]. This study, therefore, has two primary objectives. The first objective is to determine the overall effect of the environmental health policy and accumulated waste tires on deaths caused by respiratory illnesses with the given levels of the ambient pollutant concentration PM_10_ (μg/m^3^), and ozone O_3_ (pollutant concentration level expressed in ppb). Second, this study examines the impact of pollutant concentration levels PM_10_ (μg/m^3^) and O_3_ (ppb) on deaths caused by respiratory illnesses. A considerable body of epidemiologic evidence underscored the effects of exposure to PM_10_ (μg/m^3^), and O_3_ (ppb) to the respiratory system [10,12,13,21,22]. There are two reasons that this study sheds light on PM_10_ (μg/m^3^), and O_3_ (ppb). First, improvement of air quality in the concentrations of PM_10_ (μg/m^3^) is slow in spite of Taiwanese EPA efforts, while O_3_ (ppb) concentration has increased in urban areas over the past decade. Taiwan has experienced an obvious decrease in pollutants such as CO, NO_x_, and SO_2_ over the past decades and a significant upward trend of O_3_ and PM_10_ due to the characteristics of the subtropical region of Taiwan’s geographical location. Due to the data available for a 3-year moving average for a long-term evaluation and aforementioned reason, we did not include sulfur dioxide (SO_2_), nitrogen dioxide (NO_2_), and carbon monoxide (CO). The major air pollutants in Taiwan are PM_10_ (μg/m^3^), and O_3_ (ppb). Concentrations of O_3_ (ppb) are associated with odors and high temperatures that could cause respiratory illness [27], since Taiwan is located in a subtropical climate. Interestingly, the study by Ho *et al.* reported that CO (carbon monoxide) and SO_2_ (sulfur dioxide) are not positively related to asthma attacks in Taiwan [13]. The second reason for using PM_10_ (μg/m^3^) and O_3_ (ppb) as health risk factors is that they are often associated with several different respiratory illnesses. Studies on PM_10_ (μg/m^3^) with respect to respiratory illness are well documented by [19,28,29]. The literature on the connection between O_3_ (ppb) and respiratory illness is made by [27]. There are also studies that focus on the influence of O_3_ (ppb) on mortality [6,7,18].

By applying the transactional model, we investigate factors that influence health status in the community as seen in Figure 2. We evaluated: (1) subsidies provided by the environmental health policy to improve air quality; and (2) accumulated waste tires at tire recycling factories/sites by holding air pollutants, which affect respiratory disease. We hypothesize that the government’s environmental health policy will positively affect the health of the population, while accumulated waste tires at recycling factories/sites will create a negative externality (i.e., an environmental health hazard) that adversely impacts the health of the population. This takes the form of social costs by the deteriorating health status among the people in the community. In this study, we define the dependent variable of health as the number of deaths caused by bronchitis and other respiratory diseases. We note that morbidity would be an alternative measure, but the present data limitation constrains this approach [30].

We will also examine the influence of air pollution on death caused by bronchitis and other respiratory diseases. The annual average concentrations for major air pollutants in the counties and cities of Taiwan are assessed using the ambient pollutant concentration level of PM_10_ (μg/m^3^), and ozone pollutant concentration level of O_3_ (ppb) [12,20–22]. Kim *et al*. and O’Neill *et al.* employed time lags in their studies [7,18]. In our study, we will employ the time-lag variables of air pollutant, for example t-1 and t-2, since death may be affected by air pollution in the long-run [31–33]. Although the relationship between air pollution and health status would be an acute response, an increase in air pollutant concentration levels exerts an adverse effect on health status and leads to a significant loss in human capital [6,16,34]. It is essential that the effect of air pollution on public health be examined over an extended period of time. This study employed a 3-year moving average of PM_10_ and O_3_.

### 4.2. Data

The data for this study was taken from the Health and Vital Statistics annual reports conducted by the Department of Health in Taiwan from 1997 through 2002. The statistics in the reports include the number of deaths caused by bronchitis and other respiratory diseases by ICD9 classification number of basic tabulation 460–466. We also used the Annual Assessment Report of Air Pollution Control in Taiwan for the years 1999–2002. This report was created by the Department of Environmental Protection in Taiwan, and includes data for PM_10_ pollutant concentration level expressed in μg/m^3^ as well as O_3_ pollutant concentration levels expressed in ppb. The Resource Recovery, Regeneration Use, and Human Resource Inputs: Reports 1998–2002 by the Department of Environmental Protection in Taiwan includes the tire resource recovery operation revenues, as well as the quantity of waste tires collected in 15 counties and 7 large cities over four years. Data used in this study was from 1998–2001 (4-time series and 22 cross-section series).

The data suggests that the structure of the disturbance is complex, since the disturbance is assumed to result in part from the effects of omitted explanatory variables. This difficulty arises because the term of disturbance is likely to consist of time-series related disturbances, cross-section disturbances, and a combination of both [31,32]. Our particular stressor depends on the quantity of accumulated waste tires and the concentration levels of PM_10_, and O_3_ as seen in Figure 2. Thus, a straightforward functional form for this study is:

(1) $$\text{H}\;=\;f\;(\text{SUBSIDY},\;\text{Waste}\;\text{tires},\;{\text{PM}}_{10},\;{\text{O}}_{3},\;\text{D})\;+\;\varepsilon_{\text{it}}$$

where ε_it_ = u_i_ + v_t_ + e_it_, u_i_ ~ N(0, σ_u_^2^), is the cross-section error component; v_t_ ~ N(0, σ_v_^2^), is the time-series error component, and e_it_ ~ N(0, σ_e_^2^) is the combined error component. In order to mitigate the above difficulty, we applied the weighted least-square estimation procedure for maximizing the log-likelihood function [31,33] and used the population of county/city as the weight. For the above stated reasons, a log transformation is used for the analysis [32]. H represents health status which is determined by the number of deaths caused by bronchitis and other respiratory illnesses. In this study, we used death caused by bronchitis and other respiratory illnesses in persons per county/city per year by ICD9 classification number of basic tabulation 460–466. SUBSIDY denotes the amount of government subsidy issued, and we employed the government subsidy for the environmental health policy for the resource recovery program in 10,000 Taiwan dollars per county/city per year as a proxy measure for the government’s subsidy (See the detail subsidy mechanism in section of Background). To determine what the optimal level of government subsidy is, a quadratic term to describe it was used. This was done in order to evaluate the diminishing marginal effect and to determine the optimal level for the subsidy in order to decrease death caused by bronchitis and other respiratory diseases. For the variable of waste-tires in the functional form (1), we looked at the quantity of waste tires collected in tons per county/city per year as a proxy measure of accumulated waste tires at tire recycling factories and sites with time lags that will become a source of the environmental health hazard and has an influence on loss of human health in the long run [34]. Scrap and waste tire piles are not treated as hazardous materials, but once a fire occurs, the tires break down into hazardous compounds and often affect environmental pollution, and consequently exert an immediate adverse effect on human health [2,35]. Accumulation of waste tires continuously not only contributes to the environmental health hazard in a community, but is also associated with an individual’s perceptions about adverse health effect of environmental hazard [6,7,18].

The risk of air pollution, denoted by STRESSOR, includes two pollutants: PM_10_ (respirable particle pollutant concentration expressed in μg/m^3^) and O_3_ (ozone pollutant concentrations expressed in ppb). By using 1- and 2-year-lags of ozone pollutant concentration expressed in ppb in 3-year moving average, and 1- and 2-year-lags of respirable particles pollutant concentration expressed in μg/m^3^ in 3-year moving average by county/city, we are able to understand the health outcome that stems from PM10 and O_3_ in Figure 2.

D includes a city dummy variable and a year time dummy variable. The large city dummy variable represents economic activities. For the year dummy variable, the EPA promulgated the Air Pollution Control Act in 1999. Regulations have been implemented by the EPA requiring proper management. Tires should not be sent to illegal incinerators or landfills, but rather should be extracted, collected and sent to a recycling center for treatment. Industrial and institutional boilers must be permitted to ensure that any air emission from the industrial energy use is within allowable limits. The potential emission from tire derived fuel should be in a well-designed, operated, and well-maintained combustion devices. Using 1998 as the base year, allows us to examine the influence of the Air Pollution Control Act of 1999 on the population’s health. Year dummies, where the earliest year in the sample was chosen as the base year, were included for the weighted least-square procedure [31,33].

The weighted least-square estimation procedure of the maximizing log-likelihood approach was used for all of the analyses. It did not detect any serious heteroskedasticity or serial correlation. White heteroskedasticity test showed that the observations multiplied by R-squared is equal to 44.74 for RES&BRO, and 445.75 for RESPIRATORY. The 5% critical Chi-squared value for 87 degrees of freedom is 107.52, the 10% critical value is 102.07. On the basis of the White test, there is no heteroskedasticity. The Durbin-Watson statistics are 1.994 for RES&BRO, and 1.969 for RESPIRATORY. The White heteroskedasticity test indicated that the observations multiplied by R-squared is equal to 44.74 for RES&BRO, and 445.75 for RESPIRATORY. The 5% critical Chi-squared value for 87 degrees of freedom is 107.52, the 10% critical value is 102.07. On the basis of the White test, there is no heteroskedasticity. An extension of our analytical limitation indicated a need for future studies using STATA, which provides advanced methods for pooling cross-section and time-series data, such as fixed or random effect methods.

## 5. Empirical Results

Table 1 summarizes the definitions of variables and their descriptive statistics while Table 2 represents the regression results of equation (1). The most notable are the coefficients of the two variables SUBSIDY and SUBSIDY^2^ (the government subsidy for environmental health policy to increase the quality of air) in the column of RES&BRO (death caused by bronchitis and other respiratory diseases) are statistically significant.

The results in Table 2 indicate that the relationship between RES&BRO (death caused by bronchitis and other respiratory diseases) and SUBSIDY has a diminishing marginal effect of government subsidies on the number of deaths caused by bronchitis and other respiratory diseases.

Thus, based on these results we estimate that the optimal level of subsidy is about 3.22 million Taiwan dollars per county/city per year. To determine an optimal level of government subsidy, this study applied a quadratic term to a linear relationship. RES&BRO = β_0_ + β_1_ SUBSIDY + β_2_ SUBSIDY^2^. Therefore, SUBSIDY* = β_1_/(–2β_2_). The regression results show β_1_ = −0.00028 and β_2_ =0.00000004401. SUBSIDY* = (−0.00028)/(−2×0.00000004401) = 3215.91 > 0. The semi-log function does not alter the result and 3215.91 multiplied by 1,000 Taiwan dollars are about 3.22 million Taiwan dollars.

There is a lack of evidence of the impact the subsidy has on the health of the population. Namely, the deaths caused by bronchitis and other respiratory diseases (RES&BRO). We found that a ten percent increase in the subsidy would decrease death by 0.58% (about 335 persons) per county/city per year. The elasticity of death caused by RES&BRO with respect to the government subsidy (SUBSIDY) in the semi-log function is:

(2) $$\varepsilon =(\frac{\Delta \text{RES}\;\&\;\text{BRO}}{\Delta \text{SUBSIDY}}•\frac{1}{\text{RES}\&\text{BRO}})\times \text{mean}\;\text{of}\;\text{SUBSIDY}$$

and ɛ_SUBSIDY_ = −0.00028 × 646.44 = −0.134459, and ɛ_SUBSIDY_^2^ = 0.00000004401×1,746,200 = 0.07685.

Therefore, the net elasticity is ɛ_SUBSIDY_ + ɛ_SUBSIDY_^2^ = −0.057609. It is about −0.058. Furthermore, in order to balance out this health outcome, the Taiwanese government should increase the subsidy to the optimal amount of 3.22 million Taiwan dollars per county/city per year on average. The increase is needed because results show that the marginal benefit of saving lives from various respiratory illnesses is much greater than the cost of the government subsidy for environmental health policy. This can be accomplished by holding the influence of PM_10_ and O_3_ on health status constant. Deaths caused by respiratory diseases are affected by other factors with a given level of PM_10_ and O_3_. The government as a moderator will be required to specify a budget allocation to improve the health of the population through an environmental health policy aimed at recycling waste tires for efficient resource use policy.

Accumulation of waste tires at tire recycling factories and sites has a positive impact on the deaths caused by respiratory diseases and is statistically significant as seen in Table 2 (see the detail discussion on waste tires in section of Method). A ten percent increase in the amount of accumulated waste tires at tire recycling factories and sites based on their collection will lead to a 0.5% (about 101 persons) increase in the deaths per county/city per year with given the level of the ambient pollutant concentration PM_10_ (μg/m^3^), and ozone O_3_ (pollutant concentration level expressed in ppb). A government subsidy for environmental health policy that improves air quality through a tire resource recovery program will reduce the number of deaths caused by various respiratory diseases. The accumulated waste tires at tire recycling factories and sites, on the other hand, will result in deaths caused by respiratory related diseases. Our results indicate that the net effect of both the subsidy and accumulated waste tires saves two hundred thirty-four people from respiratory illnesses per county/city per year on average. However, since the current level of subsidy is far below the optimal level, any increase in subsidy towards the optimal level coupled with strict enforcement and guidance will spur a reduction in the number of deaths caused by respiratory diseases.

For the RESPIRATORY dependent variable, deaths caused by respiratory diseases in Table 2 show similar results. A ten percent increase in government subsidy for improving air quality through the tire resource recovery program leads to a 1.1 percent decrease in the number of deaths caused by respiratory diseases. The method of calculation for the combined elasticity of SUBSIDY and SUBSIDY^2^ of 1.1% in the RESPIRATORY regression is based on equation (2). The impact of the subsidy on death caused by respiratory disease is 0.52 percentage points larger than the impact on respiratory disease (1.1% (regression of RESPIRATORY) − 0.58 % (regression of RES&BRO) = 0.52 %). Results for the variable Waste tires (the accumulated waste tires at tire recycling factories/sites based on collection of waste tires) indicate that the stressor erodes the health of the population. Tire-recycling companies actually create a social cost, stemming from negative externalities to society through their economic activities. Figure 2 of the applied version of the transactional model clearly and explicitly shows that the direction of the environmental health hazard (stressor) is negatively associated with the health outcome of the population in a said community.

The level of ambient pollutant concentration PM_10_ (μg/m^3^), and the pollutant concentration level of ozone O_3_ (ppb), PM_10,t-1_ (STRESSOR) is statistically significant for both RES&BRO and RESPIRATORY. This finding indicates that an increase of one level in μg/m^3^ of PM_10_ increases the amount of deaths caused by respiratory diseases per county/city per year, which is about four hundred eighty-six deaths.

Table 2 indicates that the interacting dummy variables in a large city with air pollutants, D. O_3 t-1_ (STRESSOR), and D. O_3, t-2_ (STRESSOR) increase the number of deaths caused by respiratory related diseases in the larger and denser cities. D. PM_10, t-2_ (STRESSOR) has similar effects on the deaths caused by respiratory disease. However, the risk of the stressor D. PM_10,t-1_ (STRESSOR) lowers the amount of respiratory related fatalities in larger and denser cities. Ho *et al.* found a negative effect of the pollutant, sulfur dioxide, on the prevalence of asthma and asthma attacks [13]. That study does not, however, differentiate population density. A possible cause for the negative influence of D.CITY is better accessibility to healthcare services in large cities, thus reflecting on improving health status and outcomes [36]. By comparing the year of 1998 as a base year to 1999, 2000 and 2001, we attempt to clarify what effect the implementation of the Air Pollution Control Act in 1999 has on the health of the population. The non-significant statistical results of this study show that the impact of the policy on deaths caused by respiratory diseases is nil, but all of the coefficients are negative.

## 6. Discussion

There is an increasing interest in evaluating the environmental health policy as a means to control human health. However, there is little research pertinent to the effects of waste tires and government environmental health policy to improve air quality on the health of the population, especially in respect to respiratory illness and related mortality. Thus, it is very difficult to directly compare our results to previous studies. Our results indicate that the influence of accumulated waste tires at tire recycling factories and sites is congruent with the study by [2] which emphasized the health risks heavy metal pollution from waste tires in the soil have. The theoretical application of the Transactional Model in our study is a useful tool to evaluate the government environmental policy and the health status of the population in Taiwan. Taiwan’s environmental health policy combined with the national level of resource recovery presents a double-edged sword. The empirical results show that subsidies provided through the environmental health policy to improve air quality through the tire resource recovery program improves the health status of the population by reducing the number of deaths caused by respiratory disease. On the other hand, accumulated waste tires at tire-recycling factories and sites are needed to more effectively manage waste tires from the standpoint of environmental health policy. Unregulated storing, decomposition, and disposal processes of waste tires by inadequate compulsory regulation create environmental health hazards (i.e. disposal of used car tires, the dumping of scrap tires, incineration, stockpiles, *etc.*). The subsidy encourages tire-recycling companies to increase their economic activities under the profit base. Waste tire resource recovery is a perfect example of this trend. Economic activity by tire-recycling companies imposes social costs by way of negative externalities such as air pollution. The social cost is mainly attributable to the erosion of the population’s health in the vicinity in which these economic activities are conducted.

For the particulate matter, the findings from our study are consistent with the following studies: Samet and Pope, Vassanadumrongdee *et al.*, and Pope and Burnett, stating that health risks related to the exposure to particulate air pollution may increase mortality and shorten life expectancy [30,37,38]. The model and quantitative approaches of previous studies are different from our approach since our finding highlights the qualitative similarity of the risks posed by PM_10_ and O_3_ to the health of the population. According to our study, increases in the concentration level of pollutants, such as PM_10_ (respirable particles) and O_3_ (ozone), is associated with various respiratory diseases. Therefore, the Taiwanese government should be required to take appropriate action in communities where the air is highly polluted and it deteriorates the community’s health, as shown in Figure 2. Balancing or reducing the health hazard will result in great expenses for the government as a moderator.

The results of our study have interesting policy implications. We found that the optimal amount for the subsidy is 3.22 million Taiwan dollars per county/city per year, which is far below the current average of 0.65 million Taiwan dollars. The net effect of both a ten percent increase in subsidy and in the amount of accumulated waste tires on the number of deaths caused by respiratory diseases is to save two hundred thirty-four lives per county/city per year. An increase in general management efficiency of the tire-recycling program through effective policy and enforcement will decrease the number of respiratory diseases seen in the population. We acknowledge that we are controlling the following explanatory variables: air pollution, a geographic factor, a policy factor in our model with the statistically significant F statistics and a good level of the coefficient of determinations in our regression results. It is important to note, however, that the estimation may be overstated because respiratory diseases may be influenced by other causal factors. In Table 2, the coefficient of determinations in our regression results also show that about seventy to eighty-five percents of variations in death caused by respiratory and bronchitis related diseases are explained by the explanatory variables with F tests at 1% level that we employed. Figure 2 shows that government subsidies through environmental health policy that are intended to improve the quality of air are crucial in order to improve the population’s health. The waste-tire resource recovery program is tantamount to the environmental health policy. Revenue from the prepaid tire-recycling fee is utilized to subsidize both policies. The current source of financing seems to be causing the health disparity in question.

## 7. Conclusions

The tire-recycling program is economically important for effective resource recovery since Taiwan’s resources are limited. In the long-term environmental management perspective, the disposal of scrap and waste tires would result in serious environmental degradation requiring the policy guidance of the tire-recycling program. The findings in this study indicate that in order to improve air quality, the Taiwanese government will have to improve the health of the population by reducing the number of deaths caused by respiratory disease. On the other hand, the environmental health hazard caused by accumulated waste tires at the tire-recycling factories and sites could actually impair the health of the population and increase healthcare expenditures in Taiwan.

The purpose of this study is to establish a financing regime for the environmental health policy through the Taiwanese EPA, by imposing a tire consumer fee on motorists since they may potentially produce negative externalities for the general population. An appropriate set of taxes on tire consumers to internalize the negative externalities they create is a viable policy option. For Pigouvian taxes with negative externality or Lindahl prices with public goods, a discussion about the internalization of external costs and the socially optimal amount of pollution is beyond the scope of this study. In addition an increase in the current prepaid tire-recycling fee on tire producers and importers is not an optimal taxation policy because they do not actually produce negative externalities at this level of business activity in a competitive market. The revenue generated from the tire consumer tax from motorists could be used to provide community healthcare education as well as services for people who suffer from air pollution caused by tire recycling activity. Currently, the tire-recycling program with its unregulated storing, decomposition and disposal processes and administrative regulation causes environmental pollution. Thus, the tire consumer tax revenue could be applied to a strict pollution auditing enforcement and management guidance module for tire recycling factories.

To guide a new excise tax policy, a future study should be conducted concerning the cost/benefit effects on motorists, increases in tire recycling activities, benefits from an improvement of health outcome, as well as decreases in the loss of human resources. A future study should use morbidity variables of respiratory disease instead of using death variables in addition to including explanatory variables such as educational level, life style, and healthcare service providers and facilities as the supply side factors in a simultaneous model. These future studies will contribute to the understanding of the overall effects of the Taiwanese Government’s environmental health policy and how the policy creates adverse health effects on the people in a given community.

## Figures and Tables

**Table 1.** Definition of Variables and their Descriptive Statistics.

Variable	Mean**	Standard Deviation**
**Dependent Variables:**		
▪ RES & BRO as health status: Number of deaths caused by bronchitis and other respiratory diseases in persons per county/city per year by ICD9 classification number of basic tabulation 460–466	576.85	341.66
▪ RESPIRATORY as health status: Number of deaths caused by respiratory related disease in persons per county/city per year by ICD9 classification number of basic tabulation 460–466	487.99	293.51
**Independent Variables:**		
▪ SUBSIDY: The government subsidy to the environmental health policy for the resource recovery program in 10,000 Taiwan dollars per county/city per year*	646.44	1159.14
▪ SUBSIDY^2^: A quadratic term of the government subsidy*	174.62E+4	581.02E+4
▪ Waste tires (STRESSOR): Accumulated waste tires at tire recycling factories/sites based on collection of waste tires in tons per county/city per year	202.01	362.23
▪ O_3, t-1_ (STRESSOR): 1-year-lag level of ozone pollutant concentration expressed in ppb in 3-year moving average of the 8^th^ highest by county/city	115.24	22.50
▪ O_3, t-2_ (STRESSOR): 2-year-lag level of ozone pollutant concentration expressed in ppb in 3-year moving average of the 8^th^ highest by county/city	114.34	22.41
▪ PM_10, t-1_ (STRESSOR): 1-year-lag level of respirable particle pollutant concentration expressed in μg/m^3^ in 3-year moving average of the 8^th^ highest by county/city	139.91	35.35
▪ PM_10, t-2_ (STRESSOR): 2-year-lag level of respirable particle pollutant concentration expressed in μg/m^3^ in 3-year moving average of the 8^th^ highest by county/city	143.05	36.57
▪ D. O_3, t-1_ (STRESSOR): Interacting city dummy variable (D.CITY) with 1-year-lag level of ozone pollutant concentration expressed in ppb in 3-year moving average of the 8^th^ highest by county/city	55.06	61.01
▪ D. O_3, t-2_ (STRESSOR): Interacting city dummy variable (D.CITY) with 2-year-lag level of ozone pollutant concentration expressed in ppb in 3-year moving average of the 8^th^ highest by county/city	54.31	60.27
▪ D. PM_10, t-1_ (STRESSOR): Interacting city dummy variable (D.CITY) with 1-year-lag level of respirable particle pollutant concentration expressed in μg/m^3^ in 3-year moving average 8^th^ highest by county/city	65.18	73.65
▪ D. PM_10, t-2_ (STRESSOR): Interacting city dummy variable (D.CITY) with 2-year-lag level of respirable particle pollutant concentration expressed in μg/m^3^ in 3-year moving average of the 8^th^ highest by county/city	66.89	75.66
▪ D. CITY: A large city dummy variable, 1=if the population density is more than 1,000 per square km, otherwise 0.	0.45	0.50
▪ TIME dummy 1999: Year dummy variable, with a 1 assigned to the year of 1999 and a 0 to other year. Year of 1998 is excluded as an omitted year	0.25	0.44
▪ TIME dummy 2000: Year dummy variable, with a 1 assigned to the year of 2000 and a 0 to other year. Year of 1998 is excluded as an omitted year	0.25	0.44
▪ TIME dummy 2001: Year dummy variable, with a 1 assigned to the year of 2001 and a 0 to other year. Year of 1998 is excluded as an omitted year	0.25	0.44
▪ POPULATION: Population of county/city	101.38E+4	799.00E+3

* For the subsidy from EPA a waste-tire collection company receives T$0.6/kg from the tire-recycling company. The money basically comes from the EPA foundation through the tire-recycling company. The tire-recycling company receives T$3.2/kg, which tires are collected within the responsible district, or T$2.4/kg, which waste tires are collected outside, from the EPA foundation after the machine-comminute powder or pyrolysis (heat dissolving) process.

** The means and standard deviation are based on the total sample size of 88 (N=22 in 1998, N=22 in 1999, N=22 in 2000, and N=22 in 2001). The statistics are before log transformation (see log independent variables in Table 2).

**Table 2.** Regression Results: Evaluation of Environmental Health Policy in Taiwan.

Independent Variable	RES & BRO		RESPIRATORY	

	Estimate	t-statistics	Estimate	t-statistics

Constant	3.592a	5.733	3.272a	5.159
SUBSIDY	–2.800E-04b	–2.353	–0.306E-03b	–2.507
SUBSIDY^2^	4.401E-08b	2.315	4.745E-08b	2.465
Waste tires (STRESSOR) (ln)	0.050c	1.815	0.061b	2.221
O_3, t-1_ (STRESSOR) (ln)	–0.316	–0.520	–0.360	–0.586
O_3, t-2_ (STRESSOR) (ln)	–0.119	–0.194	–0.150	–0.243
PM_10, t-1_ (STRESSOR) (ln)	1.158c	1.903	1.422b	2.308
PM_10, t-2_ (STRESSOR) (ln)	–0.225	–0.388	–0.391	–0.666
D. O_3 t-1_ (STRESSOR)	0.034a	2.939	0.034a	2.856
D. O_3, t-2_ (STRESSOR)	0.019c	1.823	0.019c	1.782
D. PM_10, t-1_ (STRESSOR)	–0.026b	–2.051	–0.029b	–2.246
D. PM_10, t-2_ (STRESSOR)	0.018	1.556	0.021c	1.770
D. CITY	–5.796a	–5.023	–5.687a	–4.868
TIME dummy 1999	–0.112	–1.051	–0.127	–1.178
TIME dummy 2000	–0.141	–1.276	–0.143	–1.282
TIME dummy 2001	–0.128	–1.021	–0.158	–1.249

Number of observations 88			88	

Log-likelihood	–34.588		–35.670	
Multiple R	0.857		0.852	
R Square	0.734		0.725	
Adjusted R Square	0.678		0.668	

F statistics	13.238a		12.670a	

Independent variables of (ln) denote the natural logarithm. Dependent variables, RES&BRO and RESPIRATORY, are the natural logarithm. Significance of t-statistics is indicated by the following: “a” at the 1% level, “b” at the 5% level, and “c” at the 10% level. TIME dummy 1998 is an omitted variable to evaluate the implementation of the new tire recycling regulation since 1999. Another regression included the variable of population density that was not statistically significant and showed similar results with lower F statistics. The results are available from the author on request.
